# Good stress or bad stress? An empirical study on the impact of time pressure on doctoral students’ innovative behavior

**DOI:** 10.3389/fpsyg.2024.1460037

**Published:** 2024-11-08

**Authors:** Xin Zhang, Zhixing Zhao, Jie Sun, Jiajia Ren

**Affiliations:** ^1^School of Marxism, Sichuan Normal University, Chengdu, China; ^2^Sichuan Institute of Higher Studies in Culture and Education, Sichuan Normal University, Chengdu, China; ^3^XuZhou Clinical School of Xuzhou Medical University, Xuzhou Central Hospital, Xuzhou, China

**Keywords:** challenge time pressure, hindrance time pressure, innovative behavior, research self-efficacy, supervisor support

## Abstract

In recent years, with rapid societal advancement and profound transformations in knowledge production, doctoral students are increasingly facing significant time pressures. These pressures not only stem from an escalation in research tasks but also from urgent demands for innovative outputs. Grounded in Affective Events Theory, this study explores the dual impact of time pressure on the innovative behaviors of doctoral students in China. It specifically examines how challenge and hindrance time pressures affect doctoral students’ innovative behavior through the mediating role of research self-efficacy and the moderating role of supervisor support. This research employed SPSS 26.0 and Mplus 8.3 for statistical analysis, analyzing multi-time point data collected from 452 Chinese doctoral students between May and August 2023. The results reveal that challenge time pressure significantly positively impacts doctoral students’ innovative behavior, while hindrance time pressure has a significant negative impact. Furthermore, research self-efficacy partially mediates the relationship between both challenge and hindrance time pressures and innovative behavior. In this process, the moderating role of supervisor support is significant, enhancing the positive effects of challenge time pressure and mitigating the negative impacts of hindrance time pressure, highlighting the importance of supervisor support in optimizing the impact of time pressure and promoting doctoral students’ innovative behavior. These findings not only enrich the theoretical framework in the field of time pressure research but also provide practical guidance for universities and supervisors on how to support doctoral students in effectively managing time pressure and fostering their innovation.

## 1 Introduction

In the contemporary field of higher education, doctoral education represents the pinnacle of training for innovative talents, playing a crucial role in advancing the frontiers of knowledge and technological innovation. Research indicates that doctoral students’ innovative behaviors are key drivers of their academic and professional success (Tang et al., 2020; Horta, 2022). However, against the backdrop of rapid technological advancements and intensified global academic competition (Buddeberg and Hornberg, 2017; Sellar and Cole, 2017; Bennett and Burke, 2018), doctoral students often face dual pressures related to academic and career development.

Time pressure, a pervasive phenomenon in modern life and work, exerts complex effects on individuals’ innovative behaviors. Studies show that time pressure can either stimulate innovation (Ohly and Fritz, 2010; Noefer et al., 2009) or inhibit innovative activities (Hon and Lui, 2016; Maqbool et al., 2019), and may even display an inverted U-shaped effect (Baethge et al., 2018; Montani et al., 2020). Although widely studied in the occupational context, the specific impacts within higher education, particularly in doctoral education, remain underexplored.

This research, grounded in Affective Events Theory (AET) (Weiss and Cropanzano, 1996), employs survey methods and Structural Equation Modeling to delve into the mechanisms through which time pressure impacts doctoral students’ innovative behavior. The study focuses on the role of research self-efficacy as a mediating variable and the influence of supervisor support as a moderating variable. By distinguishing between challenge and hindrance time pressures, this study aims to reveal how different types of time pressures influence doctoral students’ research confidence and thereby affect their innovative behavior. Furthermore, the research emphasizes the crucial role of supervisor support in modulating the effects of time pressure, providing a comprehensive understanding of doctoral students’ innovation dynamics under stress. This research framework not only deepens our knowledge of the factors influencing doctoral students’ innovative behavior but also offers practical guidance for higher education administrators on how to stimulate doctoral students’ innovative potential in pressured situations. By systematically examining the complex interactions among these key variables, this study will provide vital theoretical insights and practical implications for optimizing the training environment for doctoral students and enhancing their capacity for innovation.

The theoretical contributions of this study are manifest in several key areas: First, it expands the research scope concerning factors influencing doctoral students’ innovative behaviors, with a particular focus on time pressure. By distinguishing between challenge-induced and hindrance-induced time pressures, the study provides a detailed analysis of their differing impacts on doctoral students’ innovation. Second, it validates the mediating role of research self-efficacy in the relationship between time pressure and innovative behaviors, enhancing the existing literature’s understanding of this mechanism. Third, the study explores the boundary conditions of supervisor support in the effects of time pressure on innovative behaviors, revealing its moderating role under different types of time pressures.

## 2 Literature review and research hypothesis

### 2.1 Challenge and hindrance time pressures and doctoral student innovative behavior

During their academic journey, doctoral students must not only fulfill academic requirements within a set timeframe but also maintain competitiveness in the fierce academic and job markets. This competition is manifested in the need to complete research projects on time, submit journal articles, prepare for academic conferences, and write dissertations, as well as the pressures associated with future career planning (Xu and Grant, 2020; Huang, 2021; Horta and Li, 2023). Faced with these multiple tasks and deadlines, doctoral students often experience intense time pressure (Maruping et al., 2014; Van Tienoven et al., 2023), which can disrupt their learning and research activities in the short term and potentially affect their innovation capacity and career trajectories in the long run.

Moreover, sustained time pressure can also negatively impact doctoral students’ mental health and work-life balance. According to the Research Mental Health Observatory Manifesto (ReMO), health-related issues faced by students during their studies are increasingly receiving attention. Prolonged time pressure may induce symptoms such as anxiety, depression, and burnout, further affecting their academic performance and quality of life (Schilbach et al., 2023). Additionally, the lack of work-life balance presents a significant challenge for doctoral students (Devonport and Lane, 2014), potentially leading to conflicts between personal life and academic development, thereby exacerbating the negative effects of time pressure.

The challenge-hindrance time pressure model developed by Chong et al. (2011) offers an effective framework for understanding the diversity of time pressures and their impacts on individuals. This model distinguishes two fundamentally different types of time pressure: challenge time pressure and hindrance time pressure, each having distinct effects on individuals’ mindset and behavior. Challenge time pressure is defined as the positive stress experienced by an individual when facing time-sensitive situations due to challenging factors such as the importance, urgency, and complexity of tasks. This type of pressure is often seen as a motivator that promotes personal growth and skill enhancement (Maruping et al., 2014; Prem et al., 2017). For example, when facing a critical project deadline, an individual might experience challenge time pressure but also regard it as an opportunity for personal achievement and career advancement. Thus, under challenge time pressure, individuals tend to demonstrate strong initiative and motivation, actively seeking solutions and ways to overcome difficulties (Zhang X. et al., 2022). In contrast, hindrance time pressure stems from factors perceived by individuals as barriers to personal development and growth, such as conflicting demands, time encroachments, and unforeseen uncertainties (Podsakoff et al., 2007). This type of pressure typically elicits negative emotions and attitudes since it threatens the individual’s ability to complete current tasks and affects long-term goals and career plans (Baethge et al., 2019; Li et al., 2023). When facing hindrance time pressure, individuals might adopt avoidance strategies to mitigate the adverse effects, although these strategies can harm their productivity and innovative potential.

Affective Events Theory (AET) highlights how stressors profoundly affect individuals’ emotions and behaviors (Rodell and Judge, 2009; Cropanzano et al., 2017). In the volatile realm of scientific innovation, doctoral students frequently encounter challenges and obstacles that elicit strong emotional responses, influencing their behavior significantly. Time pressure, a common element in academia, impacts doctoral students’ innovative actions in complex ways. It acts as a “double-edged sword” (Zhang X. et al., 2022), where on one side, the urgency from challenging tasks can enhance engagement and drive innovation (Van Tienoven et al., 2023). On the other side, hindrance pressures from conflicts between personal and academic responsibilities reduce control and self-confidence, leading to defensive behaviors that stifle innovation (Ashencaen Crabtree et al., 2021).

Based on this, the study proposes the following hypotheses:

*H1a*: Challenge time pressures have a significant positive impact on doctoral students’ innovative behavior.

*H1b*: Hindrance time pressures have a significant negative impact on doctoral students’ innovative behavior.

### 2.2 The mediating role of research self-efficacy

Affective Events Theory emphasizes the mediating role of emotions between work events and attitudinal behaviors (Weiss and Cropanzano, 1996). According to this theory, self-efficacy as a core psychological mechanism significantly influences how individuals perceive, interpret, and respond to various pressures and emotional challenges in work and learning environments (Pressley and Ha, 2021; Hu et al., 2024). This theoretical framework is particularly applicable in higher education, where self-efficacy directly affects students’ emotional responses and behaviors in facing complex learning tasks (van Dinther et al., 2011; Bartimote-Aufflick et al., 2016).

Research self-efficacy, as a specific application of Albert Bandura’s self-efficacy theory (Bandura, 1982) in the domain of scientific research, focuses on researchers’ confidence in their ability to complete research tasks (Forester et al., 2004). This confidence encompasses not only a self-assessment of technical abilities but also psychological preparedness for the inevitable challenges and difficulties encountered during the research process. Studies have shown that research self-efficacy significantly affects researchers’ performance, research output, enthusiasm, and career aspirations (Adedokun et al., 2013; Livinƫi et al., 2021) and enhances their ability to cope with research challenges, continuous learning motivation, and resilience in the face of failure (Liu et al., 2019; Nazari et al., 2021). Specifically, researchers with high levels of research self-efficacy are more likely to adopt proactive strategies to overcome obstacles in research, such as using creative thinking and effective problem-solving strategies, rather than avoiding problems or giving up. This confidence motivates them to explore new methods and solutions when facing research challenges and to recover quickly when encountering obstacles and failures. This proactive approach directly influences the occurrence of innovative behavior. Research self-efficacy not only enhances researchers’ problem-solving capabilities but also boosts their motivation and efficiency during the research process. Therefore, a high level of research self-efficacy makes researchers more likely to adopt innovative approaches, thereby positively impacting their research outcomes and overall research performance.

Time pressure is a common phenomenon in scientific activities, typically caused by looming project deadlines, funding use limitations, or expectations for academic publication. Additionally, doctoral students often need to assume multiple roles (such as students, employees, teachers) and multiple responsibilities (such as conducting research, teaching, attending conferences, etc.), which together increase the perception of time pressure (Winstone and Moore, 2017; Glorieux et al., 2024). According to the Affective Events Theory, different situational factors (such as challenge and hindrance time pressures) have different impacts on individuals’ emotional experiences (Chacko and Conway, 2019). Challenge time pressure is often seen as a motivational factor, promoting individuals to achieve their goals, while hindrance time pressure is viewed as an obstacle to achieving goals (Zhang X. et al., 2022). Specifically, challenge time pressure can inspire doctoral students to undertake more rational research and study task planning, coping with the tension of time resources. This pressure enhances doctoral students’ agency and research self-efficacy, enabling them to better handle negative emotions (Calaguas and Consunji, 2022), thereby reducing anxiety (Azizli, 2015) and promoting the emergence of innovative behaviors. In contrast, hindrance time pressure often disrupts doctoral students’ research processes, causing them to lose control over time resources, which triggers negative emotional experiences and thereby lowers research self-efficacy, inhibiting innovative behaviors.

Based on the aforementioned theoretical frameworks and empirical studies, it is postulated that research self-efficacy may play a mediating role in the process through which challenge and hindrance time pressures influence research behavior. When research self-efficacy is enhanced, doctoral students might exhibit more positive emotional states and demonstrate a stronger tendency toward innovative behavior, even under time pressure. Conversely, a decrease in research self-efficacy could potentially exacerbate the negative impact of hindrance time pressure on doctoral students, leading to a propensity to avoid challenges and innovation. This potential mediating effect underscores the significance of enhancing research self-efficacy in both managing research-related time pressure and fostering research innovation. Such a theoretical proposition provides direction for subsequent empirical investigations, particularly in exploring the specific mechanisms through which research self-efficacy may operate within the complex relationship between time pressure and innovative behavior in academic settings.

Based on this, the following hypotheses are proposed:

*H2a*: Research self-efficacy mediates the positive impact of challenge time pressure on doctoral students’ innovative behaviors.

*H2b*: Research self-efficacy mediates the negative impact of hindrance time pressure on doctoral students’ innovative behaviors.

### 2.3 The moderating role of supervisor support

Supervisors play a crucial role in the doctoral journey, being primarily responsible for the research learning and innovation process. They provide essential resources and support academically, and pay attention to emotional aspects, significantly impacting doctoral students’ enthusiasm and research effectiveness (Gu et al., 2017; Zhang Y. et al., 2022). According to Overall et al. (2011), supervisor support is defined as behaviors that allow students autonomy in decision-making, respect and encourage their views, and provide timely guidance when needed. This support is subdivided into academic, emotional, and autonomy support, each profoundly influencing the doctoral experience.

The Affective Events Theory emphasizes that situational factors, such as time pressure, are crucial sources of individual emotional experiences that significantly affect behavior and performance (Gardner, 2012). Under this theoretical framework, supervisor support, as a key shaper of the learning environment (Hughes et al., 2018), has the potential to moderate doctoral students’ psychological states and behaviors under pressure (Buirski, 2022). Studies have shown that effective supervisor support can significantly reduce anxiety in graduate students (Franke and Arvidsson, 2011) and decrease the likelihood of depression (Le et al., 2021).

Supervisor support not only provides academic guidance but also encompasses care and management of students’ emotions, especially in dealing with time pressure. Good supervisor support helps doctoral students more effectively handle challenge and hindrance time pressures, maintain or enhance their research self-efficacy, and ignite their passion for research and innovation (Shang et al., 2023). For instance, when facing tight deadlines and high standards, adequate supervisor support can transform these pressures into positive factors that enhance research self-efficacy, thereby further promoting innovative behaviors. Conversely, if supervisors fail to provide necessary support, the same hindrance pressures (such as continuous project delays or resource shortages) could become significant obstacles on the doctoral research path. If supervisors neglect students’ academic needs and emotional support, treating them as cheap labor, this not only risks alienating the student-supervisor relationship but may also exacerbate doctoral students’ anxiety and depression (Löfström et al., 2023; White et al., 2023). Such negative emotions can suppress their research self-efficacy. However, when supervisor support is high, the traditional negative effects of hindrance time pressure can be mitigated and possibly transformed into positive outcomes. Emotional and academic support from supervisors helps doctoral students view these pressures as challenges, enhance their research self-efficacy, and stimulate research innovation.

Based on these observations, the study proposes the following hypotheses:

*H3a*: When supervisor support is high, the positive effect of challenge time pressure on research self-efficacy is enhanced, and vice versa.

*H3b*: When supervisor support is high, the negative effect of hindrance time pressure on research self-efficacy is mitigated and may even become positive.

Based on the aforementioned theoretical frameworks and empirical studies, this research proposes a hypothesized moderated mediation model. In this model, supervisor support may not only moderate the relationship between challenge and hindrance time pressures and doctoral students’ innovative behavior but also potentially moderate the mediating role of research self-efficacy. Specifically, for doctoral students receiving high levels of supervisor support, the mediating effect of research self-efficacy on the relationship between challenge time pressure and innovative behavior may be more pronounced. Conversely, for those receiving low levels of supervisor support, this mediating effect might be relatively weaker. Similarly, supervisor support may influence the mediating role of research self-efficacy in the relationship between hindrance time pressure and innovative behavior. Under high levels of supervisor support, the negative impact of hindrance time pressure on innovative behavior through research self-efficacy might be significantly mitigated. This mitigation could potentially manifest as research self-efficacy transforming these negative pressures into positive drivers of innovation. This potential transformation mechanism may provide doctoral students facing pressure with more effective psychological resources and possibly stimulate their innovative potential.

Based on this, the study proposes the following hypotheses:

*H4a*: Supervisor support enhances the positive mediating effect of challenge time pressure through research self-efficacy on doctoral students’ innovative behavior.

*H4b*: Supervisor support weakens the negative mediating effect of hindrance time pressure through research self-efficacy on doctoral students’ innovative behavior.

**Figure 1:**
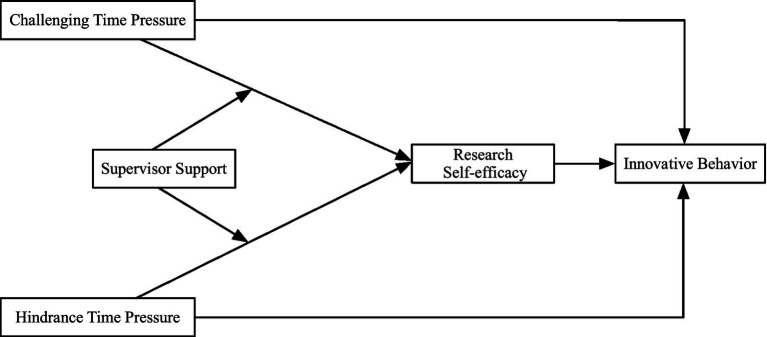
Research model.

The study constructs the theoretical conceptual model as shown in Figure 1.

## 3 Method

### 3.1 Sample and procedure

The survey sample for this study was drawn from doctoral students enrolled at 19 universities across Sichuan, Jiangsu, Zhejiang, and Guangdong provinces. The recruitment process primarily utilized two channels: first, through collaboration with graduate schools at various universities to reach potential participants, and second, by posting recruitment information on academic social networks and graduate forums. The inclusion criteria were: (1) full-time doctoral students; (2) enrolled for more than 6 months; (3) participation in the study was voluntary. Details on the distribution of the sample can be seen in Table 1. To obtain a large number of valid samples, some surveys were conducted with the assistance of university graduate departments through electronic questionnaires. To alleviate respondents’ concerns and increase the validity of the questionnaires, the surveys were designed anonymously and with sensitive information removed, and the purpose and intent of the research were communicated to participants. To reduce common method bias, data collection spanned 3 months from July to October 2023, occurring in three waves: Wave 1: Time pressure, innovative behavior, and control variables were collected, distributing 1,200 questionnaires and retrieving 816 responses, with 672 valid questionnaires, yielding an initial valid response rate of 82.352%; Wave 2: Four weeks later, the 672 valid responses from Wave 1 were followed up to collect data on research self-efficacy, retrieving 574 responses, of which 548 were valid, giving a second-wave valid response rate of 95.47%; Wave 3: Four weeks after Wave 2, the 548 valid responses were followed up to collect data on supervisor support, retrieving 455 responses, of which 452 were valid, resulting in a third-wave valid rate of 99.34%.

**Table 1:** Sample distribution.

Title	Category	Sample size	Percentage	Title	Category	Sample size	Percentage
Population statistics	Male	233	51.5%	School type	Double first-rate universities	272	60.2%
	Female	219	48.5%		General university	180	39.8%
Grade composition	Year 1	108	23.9%	Admission method	Master’s direct doctoral degree	96	21.2%
	Year 2	132	29.2%		Application for review	177	39.2%
	Year 3	105	23.2%		Common examination	179	39.6%
	Year 4	77	17.0%	Subject classification	Natural sciences	143	31.6%
	Year 5+	30	6.6%		Human sciences	309	68.4%

### 3.2 Measurement tools

To ensure the reliability and validity of the measurement tools, this study uses mature scales widely applied in authoritative journals domestically and internationally. Adhering strictly to a translation-back-translation procedure and refined through expert discussions, the scales are designed to ensure semantic accuracy and formal appropriateness in accordance with Chinese language standards. All scales use a 7-point Likert scale, where “1–7” indicates the level of agreement with the item.

#### 3.2.1 Time pressure

The scale, developed by Chong et al. (2011), includes two variables: Challenge Time Pressure (CTP) and Hindrance Time Pressure (HTP). The former comprises items such as “Completing this task on time is extremely important, making me feel time pressure” and “Completing tasks on time requires overcoming technical complexities, leading to a sense of time pressure,” totaling five items. The latter includes items like “My supervisor frequently demands updates on task completion, creating a sense of urgency” and “I feel pressured by daily task switching,” totaling eight items. Alpha coefficients are 0.888 and 0.903, respectively.

#### 3.2.2 Innovative behavior

Based on the scale developed by Scott and Bruce (1994), modified to reflect the actual circumstances of doctoral students. It includes items like “I frequently propose creative ideas in my research” and “I often communicate and promote my new research ideas to peers or supervisors,” totaling eight items. Alpha coefficient is 0.923.

#### 3.2.3 Research self-efficacy

The 4-item scale developed by Adedokun et al. (2013) was used for measurement, and the representative item was “I am confident that I have the ability to become an excellent scientific researcher”; “I have a strong interest in scientific research activities.” Alpha coefficient is 0.916.

#### 3.2.4 Supervisor support

Uses a 15-item, three-dimensional scale developed by Overall et al. (2011), comprising academic support (five items), emotional support (five items), and autonomy support (five items). Example items include “My supervisor provides advice to help me find needed resources” and “My supervisor shows respect and values me.” Alpha coefficient is 0.961.

#### 3.2.5 Control variables

Considering that innovative behavior is the result of doctoral students’ psychological states and behavioral choices, this paper controls for demographic variables such as gender, age, grade, field of study, academic level of the institution, and supervisor’s position, in line with previous research (Li et al., 2019; Luis et al., 2020; Dai et al., 2022).

### 3.3 Analytical methods

This study employed SPSS 26.0 and Mplus 8.3 for statistical analysis, chosen for their robust capabilities in handling complex statistical models. Initially, to ensure data quality, Harman’s single factor test was utilized to assess common method bias, followed by confirmatory factor analysis to test discriminant validity between variables. These methods effectively evaluate the validity and reliability of measurements. Subsequently, hierarchical regression analysis was used to preliminarily explore the relationships between variables, laying the groundwork for more in-depth analysis. The selection of Structural Equation Modeling (SEM) for testing main effects, mediating effects, and moderating effects was based on its ability to estimate complex relationships among multiple variables simultaneously, making it particularly suitable for the analysis of mediation and moderation effects involved in this study. Compared to traditional regression methods, SEM provides a better handling of measurement errors and offers more accurate effect estimates (Hayes, 2013; Abrahim et al., 2019). Additionally, the Bootstrap method was employed to generate confidence intervals, enhancing the reliability of indirect effect estimates. By repeatedly resampling to generate an empirical sampling distribution, the Bootstrap method is particularly suited to the potentially non-normal distribution data in this study, providing more precise confidence intervals and significance tests (Ghasemy et al., 2020).

The integrated use of these methods not only enhances the rigor and reliability of the analysis but also allows for a more comprehensive understanding of the complex interactions between time pressure, research self-efficacy, supervisor support, and doctoral students’ innovative behaviors. This, in turn, provides deeper insights into research within the field of higher education.

## 4 Results

### 4.1 Common source bias and confirmatory factor analysis

Since the data was collected via self-assessment by doctoral students and despite the design mitigating common method bias through clear study intent, confidentiality emphasis, and multi-wave responses, some bias remains inevitable. To ensure data integrity, Harman’s single factor test was applied, revealing that the unrotated first factor accounted for 31.405% of variance (below 40%), indicating that common method bias was not severe. Further, by integrating a common latent factor into the structural equation model, the comparison revealed no significant change in model fit after controlling for the common method factor (Δ*χ*^2^/df = 0.146, ΔRMSEA = 0.002, ΔCFI = 0.007, ΔTLI = 0.002), confirming the absence of severe common method variance, allowing for further data analysis.

Using Mplus 8.3, confirmatory factor analysis was conducted on the primary variables: Challenge Time Pressure, Hindrance Time Pressure, Innovative Behavior, Research Self-Efficacy, and Supervisor Support to evaluate discriminant validity among them. According to Table 2, the five-factor model showed good fit (*χ*^2^/*df* = 1.151, RMSEA = 0.018, CFI = 0.991, TLI = 0.99, SRMR = 0.003), significantly better than the other four alternative models, demonstrating good discriminant validity among the five main variables.

**Table 2:** Fitness indexes of scales.

	*χ* ^2^	*df*	RMSEA	CFI	TLI	SRMR
Model 1: five factor model	885.258	769	0.018	0.991	0.99	0.033
Model 2: four-factor model	2,787.663	773	0.076	0.836	0.825	0.132
Model 3: three-factor model	3,239.012	776	0.084	0.799	0.787	0.097
Model 4: two-factor model	4,479.76	778	0.103	0.698	0.681	0.124
Model 5: one-factor model	6,932.902	779	0.132	0.498	0.471	0.171

Model 2: “Challenge Time Pressure + Hindrance Time Pressure” combined into one factor; Model 3: “Challenge Time Pressure + Hindrance Time Pressure + Research Self-Efficacy” combined into one factor; Model 4: “Challenge Time Pressure + Hindrance Time Pressure + Research Self-Efficacy + Supervisor Support” combined into one factor; Model 5: All variables combined into one factor.

### 4.2 Descriptive statistics and correlation analysis

The means, standard deviations, and inter-variable correlation coefficients are shown in Table 3. Challenge Time Pressure is positively correlated with the mediating variable Research Self-Efficacy (*r* = 0.225, *p* < 0.01) and positively correlated with the outcome variable Innovative Behavior (*r* = 0.403, *p* < 0.01); Hindrance Time Pressure is positively correlated with the mediating variable Research Self-Efficacy (*r* = 0.360, *p* < 0.01) and positively correlated with the outcome variable Innovative Behavior (*r* = 0.401, *p* < 0.01); the mediating variable Research Self-Efficacy is positively correlated with the outcome variable Innovative Behavior (*r* = 0.400, *p* < 0.01). Thus, the results of the correlation analysis preliminarily support the related hypotheses.

**Table 3:** Descriptive statistics and correlation coefficient of variables.

	*M*	SD	1	2	3	4	5	6	7	8	9	10	11
1. Gender	1.480	0.500											
2. Age	2.430	0.622	−0.139^**^										
3. Grade/year	2.530	1.212	0.019	0.453^**^									
4. Discipline type	6.940	3.680	0.015	0.029	0.002								
5. University level	1.400	0.490	0.297^**^	−0.137^**^	−0.015	−0.01							
6. Supervisor position	2.180	0.759	−0.165^**^	0.120^*^	0.067	0.07	−0.161^**^						
7. Challenging time pressure	3.966	1.544	−0.077	0.09	0.009	0.057	−0.039	0.038	**0.888**				
8. Hindering time pressure	3.804	1.502	−0.134^**^	0.164^**^	0.079	−0.101^*^	−0.229^**^	0.111^*^	0.018	**0.903**			
9. Research self-efficacy	4.047	1.671	−0.158^**^	0.206^**^	0.092	0.031	−0.132^**^	0.140^**^	0.225^**^	−0.360^**^	**0.916**		
10. Supervisor support	4.117	1.383	−0.078	0.241^**^	0.135^**^	0.034	−0.133^**^	0.131^**^	0.164^**^	0.196^**^	0.524^**^	**0.961**	
11. Innovative behavior	3.913	1.419	0.125^**^	−0.07	0.009	0.043	0.207^**^	−0.099^*^	0.403^**^	−0.401^**^	0.400^**^	0.227^**^	**0.923**

*n* = 452; *p < 0.05, **p < 0.01, ***p < 0.001. Bold values are autocorrelation coefficients.

### 4.3 Hypothesis testing

#### 4.3.1 Main effect testing

**Figure 2:**
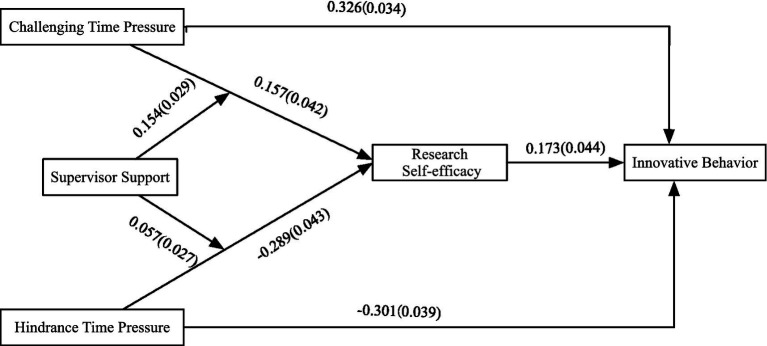
Path coefficient diagram.

The full model was tested using Mplus 8.3 as shown in Figure 2. Results indicate that Challenge Time Pressure positively influences Innovative Behavior (*r* = 0.326, *p* < 0.001), while Hindrance Time Pressure negatively influences Innovative Behavior (*r* = −0.301, *p* < 0.001). Therefore, Hypotheses 1a and 1b are supported.

#### 4.3.2 Mediation effect testing

As shown in Figure 2, Challenge Time Pressure positively affects Research Self-Efficacy (*r* = 0.157, *p* < 0.001), while Hindrance Time Pressure negatively affects it (*r* = −0.289, *p* < 0.001); Research Self-Efficacy positively influences Innovative Behavior (*r* = 0.173, *p* < 0.001), preliminarily verifying Hypotheses 2a and 2b.

Further testing of the mediation effect was conducted using the bootstrapping method with 10,000 samples. The results show that the indirect effect of Challenge Time Pressure on Innovative Behavior through Research Self-Efficacy is 0.027, with a 95% confidence interval of [0.052, 0.011]. The indirect effect of Hindrance Time Pressure through Research Self-Efficacy on Innovative Behavior is −0.050, with a 95% confidence interval of [−0.084, −0.024], supporting Hypotheses 2a and 2b.

#### 4.3.3 Moderation effect testing

As shown in Figure 2, supervisor support positively moderates the relationship between Challenge Time Pressure and Research Self-Efficacy (*r* = 0.154, *p* < 0.001), and it also positively moderates the relationship between Hindrance Time Pressure and Research Self-Efficacy (*r* = 0.057, *p* < 0.001). The moderating effects were preliminarily confirmed.

**Figure 3:**
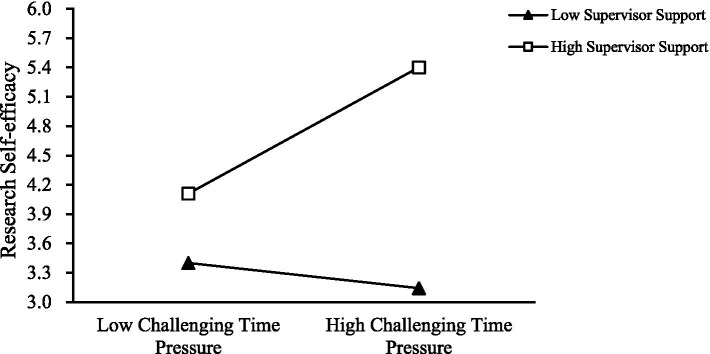
Moderating effect of supervisor support on challenging time pressure and research self-efficacy.

**Figure 4:**
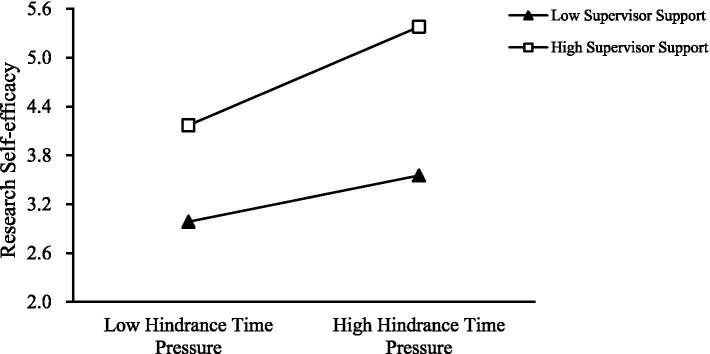
Moderating effect of supervisor support on hindrance time pressure and research self-efficacy.

To further test the moderating effect of supervisor support between Time Pressure (Challenge and Hindrance) and Research Self-Efficacy, it was found that as supervisor support increases, the positive relationship between Challenge Time Pressure and Research Self-Efficacy strengthens (as shown in Figure 3); similarly, the positive relationship between Hindrance Time Pressure and Research Self-Efficacy also strengthens with increased supervisor support (as shown in Figure 4). Thus, Hypotheses 3a and 3b are supported.

#### 4.3.4 Conditional process model testing

Using the conditional process model proposed by Hayes and Rockwood (2020), the study explored the impact mechanisms and boundary conditions of independent variables on dependent variables. To validate the moderating effect of supervisor support on the paths “Challenge Time Pressure → Research Self-Efficacy → Innovative Behavior” and “Hindrance Time Pressure → Research Self-Efficacy → Innovative Behavior,” the bootstrapping method with 10,000 samples was utilized. Table 4 shows the correlation coefficients, standard errors, and confidence intervals for these paths, indicating significant effects, thereby supporting Hypotheses 4a and 4b.

**Table 4:** Effect analysis for condition process model.

	CTP → Research self-efficacy → IB				HTP → Research self-efficacy → IB			
	Level	Estimate	S.E.	95%CI	Level	Estimate	S.E.	95%CI
Supervisor support	Low	0.018	0.016	[0.107, 0.031]	Low	0.015	0.013	[−0.108, −0.0030]
	High	0.073	0.025	[0.008, 0.033]	High	0.104	0.025	[−0.070, −0.015]
	Odds	0.054	0.023	[0.074, 0.035]	Odds	0.089	0.023	[−0.027, −0.003]

CTP, Challenge Time Pressure; HTP, Hindrance Time Pressure; IB, Innovative Behavior.

## 5 Discussion

Time pressure, as a prevalent issue in contemporary society, has widespread impacts, extensively explored within professional contexts by academics. However, within the educational realm, questions remain unanswered about whether time pressure similarly affects doctoral students, influences their innovative behaviors, and what the mechanisms and boundary conditions of its effects are.

First, does time pressure promote or inhibit doctoral students’ innovative behavior? The study reveals that the impact of time pressure on doctoral students’ innovative behavior presents a complex “double-edged sword” effect. Hypotheses 1a and 1b were confirmed, indicating that challenge-related time pressure promotes innovative behavior, while hindrance-related time pressure inhibits it. This finding not only supports existing research on the dual effects of time pressure (French et al., 2019) but also extends it to the domain of higher education. Specifically, challenge-related time pressure, such as dissertation submission deadlines, can stimulate doctoral students’ intrinsic motivation and sense of achievement, prompting them to adopt innovative approaches to meet challenges. This aligns with the research by Ohly and Fritz (2010), who found that moderate time pressure can enhance job engagement and creativity. Conversely, hindrance-related time pressure, such as cumbersome administrative tasks, may consume doctoral students’ cognitive resources, diminishing their capacity to engage in innovative activities. This result echoes the analysis by Rostami et al. (2019), which associated hindrance stressors with lower job performance and motivation. However, it is important to note that the effects of time pressure may vary depending on individual differences and contextual factors. For example, some doctoral students may thrive under pressure, while others may be more susceptible to its negative impacts. Future research could consider incorporating personality traits, such as resilience or self-efficacy, as moderating variables to more comprehensively understand the differentiated impacts of time pressure.

Second, how do doctoral students’ psychological states and reactions under time pressure influence their innovative behavior? This study, utilizing Affective Events Theory (AET), explores how time pressure can stimulate emotional and psychological responses, which in turn mediate behavioral outcomes. The findings reveal that challenge-related time pressure enhances doctoral students’ research self-efficacy by stimulating their intrinsic motivation and positive self-assessment of their research capabilities, thereby fostering innovative behavior. This aligns with the findings of Khliefat et al. (2021), who noted that challenge stressors can ignite an individual’s drive and initiative, promoting innovative problem-solving approaches. Conversely, hindrance-related time pressure increases psychological strain and reduces feelings of control, diminishing research self-efficacy and inhibiting innovative behavior. Such pressure is often perceived as a threat, not only limiting doctoral students’ ability to manage current tasks but also impeding their long-term academic and career goals. Thus, the negative effects of hindrance-related time pressure can be more pronounced, as Chuderski (2016) points out, with the detrimental impact of time pressure on learning often outweighing its motivational benefits. These findings not only demonstrate that research self-efficacy plays a pivotal mediating role in how time pressure affects innovative behavior—confirming Hypotheses 2a and 2b—but also deepen our understanding of the processes by which time pressure influences behavior, providing a theoretical basis for interventions. For example, offering appropriately challenging tasks and timely positive feedback may enhance doctoral students’ research self-efficacy, thus fostering innovative behavior. However, future research should consider additional potential mediating variables, such as job engagement or psychological capital, to build a more comprehensive theoretical model.

Third, what moderates the impact of time pressure on doctoral students’ innovative behavior? Supervisor support, a key contextual variable, plays a critical role when doctoral students face time pressure. This study investigates how supervisor support modulates the effects of challenge and hindrance-related time pressures on doctoral students’ innovative behavior. It was found that supervisor support not only enhances the positive effects of challenge-related time pressure but also significantly mitigates the negative impacts of hindrance-related time pressure, potentially transforming it into a positive force. This finding aligns with the research of Widmer et al. (2012) and Liu et al. (2022), which emphasizes that time pressure typically has both positive and negative impacts, and it does not automatically produce motivational effects. Positive outcomes only emerge when negative impacts are effectively managed under specific conditions. Therefore, the potential benefits of time pressure can only be fully realized under the right supportive and contextual boundary conditions. Specifically, the guidance, encouragement, psychological support, and problem-solving support provided by supervisors are crucial components of a positive academic environment. These supports help doctoral students better understand and manage time pressure, especially when facing complex research tasks and tight deadlines. The results indicate that under high levels of supervisor support, challenge-related time pressure can stimulate doctoral students’ research self-efficacy and encourage them to engage in innovative behaviors. In the same supportive environment, the negative impact of hindrance-related time pressure on innovative behavior is significantly reduced, allowing students to view difficulties as challenges, which in turn enhances their research self-efficacy and motivation. Hypotheses 3a, 3b, 4a, and 4b are supported. The findings are consistent with previous research, indicating that the relationship between stressors and outcome variables is moderated by organizational contextual variables (Leung et al., 2011).

## 6 Conclusions and practical implications

### 6.1 General findings

The empirical analysis yielded the following findings:

First, challenge-related time pressure has a significant positive effect on doctoral students’ innovative behavior, while hindrance-related time pressure has a significant negative effect. This aligns with the dual stress model proposed by Cavanaugh et al. (2000), where challenge stress is seen as “good” stress, and hindrance stress is considered “bad” stress (Rodell and Judge, 2009). When time pressure is perceived as a challenge, although it brings discomfort, it enhances a sense of achievement and challenge, promoting greater initiative and focus among doctoral students during their learning process, while also stimulating innovative thinking and behaviors. Conversely, when time pressure becomes a hindrance, it psychologically triggers resistance in doctoral students, thereby impeding the realization of innovative behaviors.

Second, research self-efficacy mediates the relationship between time pressure and doctoral students’ innovative behavior. This finding further validates the applicability of Affective Events Theory (AET), which emphasizes how emotions affect individuals’ self-efficacy and behaviors (Weiss, 2002). Different types of time pressure induce different emotional experiences, leading to different behavioral responses, with self-efficacy playing a crucial mediating role. Under challenge-related time pressure, doctoral students enhance their likelihood of engaging in innovative behaviors by boosting their self-efficacy to alleviate the psychological burden brought by pressure; under hindrance-related time pressure, the influence of hindrance factors may induce negative emotions, reducing their self-efficacy, and thus inhibiting innovative behaviors.

Third, supervisor support moderates the direct effects of challenge-related and hindrance-related time pressures on research self-efficacy, indicating that supervisor support is an important organizational context variable affecting doctoral students’ emotional regulation and behavioral responses (Raposa and Hurd, 2021; Wollast et al., 2023). Under challenge-related time pressure, high levels of supervisor support enhance the positive effects of research self-efficacy, thereby strengthening its promotive effect on innovative behaviors; under hindrance-related time pressure, supervisor support can significantly mitigate negative impacts, potentially transforming these pressures into motivations for innovation.

Fourth, supervisor support also moderates the influence of challenge-related and hindrance-related time pressures on doctoral students’ innovative behaviors through research self-efficacy. In the context of challenge-related time pressure, the support and encouragement from supervisors can elicit positive emotions in doctoral students, enhancing their self-efficacy and thus stimulating innovative behaviors; conversely, in an environment of hindrance-related time pressure, the absence of supervisor support may lead doctoral students to experience increased depression and anxiety, which lowers their self-efficacy and inhibits innovative behaviors. These findings are consistent with previous research, confirming the significant impact of the interaction between environmental and individual factors on doctoral students’ innovative behaviors (Acquaye and Pownall, 2021).

### 6.2 Practical implications

This study offers practical insights in the following areas:

First, enhance awareness and response to the effects of time pressure. Universities and supervisors should deeply understand the dual effects of time pressure, which can promote doctoral students’ innovative behaviors under certain conditions and inhibit them under others. Thus, higher education institutions and supervisors should take targeted measures to optimize the management and application of time pressure. Universities should develop a diversified doctoral evaluation system and set moderate and challenging research goals and tasks for doctoral students, stimulating the positive effects of challenge-related time pressure through clear research objectives and reasonable project timelines. Moreover, hindrance-related time pressures should be reduced, for example, by minimizing unnecessary administrative burdens and optimizing resource allocation to enhance doctoral students’ innovation enthusiasm. In practice, supervisors should lead by example, engaging earnestly in research, inspiring students’ passion for innovation, and considering students’ receptiveness and capabilities when assigning tasks and communicating expectations, to avoid overloading students with a uniform guidance approach.

Second, strengthen the cultivation of research self-efficacy. Universities and supervisors should pay more attention to doctoral students’ research self-efficacy and their mental health, as both are crucial for the students’ innovative behaviors and stress management capabilities. Research self-efficacy plays a mediating role between time pressure and innovative behavior, thus higher education institutions should foster doctoral students’ research self-efficacy through practical research training and increased positive feedback and recognition. Additionally, universities should regularly conduct stress monitoring surveys for doctoral students, strengthen systematic psychological service provision, and improve mental health education for doctoral students to help them enhance their self-regulation abilities and manage psychological stress effectively (McCray and Joseph-Richard, 2021). Supervisors play a key role in this process, not only providing support during mental health crises and helping students seek professional psychological counseling and treatment but also by enhancing research skills through seminars and workshops. Regular workshops on mental health and stress management should be organized to help doctoral students learn and apply effective stress coping strategies. These measures can strengthen doctoral students’ confidence in their research capabilities, thereby enabling them to better manage time pressures and discover the value and meaning of academic life.

Lastly, enhance supervisor support and emotional care. Universities should strengthen training for supervisors to enhance their support and emotional care for doctoral students. Supervisors, as the primary responsible parties for doctoral training (Almlöv and Grubbström, 2023), directly influence the emergence and exacerbation of students’ academic difficulties (Wilkin et al., 2023). Effective supervisor support can significantly enhance the positive effects of challenge-related time pressure and alleviate the negative impacts of hindrance-related time pressure. Therefore, universities should focus on building management and academic support systems for supervisors, ensuring they can provide necessary research guidance and psychological counseling. Specific measures include training supervisors to enhance their psychological counseling skills, enabling them to support students emotionally while also providing research guidance. Moreover, supervisors should be encouraged to respect students’ academic interests and feelings and to develop personalized training plans based on students’ personalities and characteristics. Universities should also institutionalize activities such as group meetings, lecture forums, and academic conferences to enhance doctoral students’ academic capabilities and alleviate pressures related to research self-efficacy. By establishing strong student-supervisor relationships and providing daily life care and emotional support during studies and research, supervisors can help students better handle the challenges of time pressure, enhance their research confidence, and improve their academic happiness. These measures, working together, can effectively motivate doctoral students’ innovative behaviors and improve their overall academic performance.

### 6.3 Limitations and future research

Despite its rigorous design and valuable insights, this study has limitations that provide directions for future research.

Although multi-time point data collection was used, all variable measurements in this study rely on doctoral students’ self-reports, which may be subject to common method bias. Future research could use diary study methods or field surveys, which collect more real-time data from daily life and actual work scenarios to provide more accurate and specific behavioral performances and reduce self-report biases.

Secondly, while this study explored the mediating role of research self-efficacy, other potential psychological mechanisms, such as psychological exhaustion and resilience, may also play roles between time pressure and doctoral students’ innovative behaviors. Future studies could consider including these variables to construct a more comprehensive mediation model to delve deeper into the complex psychological processes affecting doctoral students’ innovative behaviors.

Lastly, the sample of this study was solely from China, which may be influenced by specific cultural backgrounds. Future research should consider cross-cultural samples to explore whether there are differences in behaviors and reactions among doctoral students from different cultural backgrounds, to enhance the universality and transferability of the research findings.

## Data Availability

The raw data supporting the conclusions of this article will be made available by the authors without undue reservation.

## Appendix

### Time pressure

#### Challenge time pressure

1. Completing this task on time is crucial and makes me feel a sense of urgency.
2. Completing this task on time requires the help of other students, which makes me feel the pressure of time.
3. Completing this task on time requires collective effort, which makes me feel a sense of urgency.
4. Completing learning tasks on time requires overcoming difficult and complex technical problems, which makes me feel time-pressured.
5. The urgent need to successfully complete the task gives me a sense of time pressure.

#### Hindrance time pressure

6. Realizing I cannot complete the task on time makes me feel a sense of urgency.
7. Being unable to further improve the final outcomes through upgrades makes me feel time-pressured.
8. My supervisor’s frequent demands for progress updates make me feel pressured.
9. Constant changes to the task give me a sense of urgency.
10. Insufficient buffer time for completing the task creates a sense of urgency.
11. Switching between different tasks daily puts significant time pressure on me.
12. Continuous high-intensity work puts time pressure on me.
13. The urgency of the task disrupts the balance of my personal life.

#### Innovative behavior

14. I often come up with creative ideas and concepts in my research.
15. I frequently communicate and promote my new research ideas to peers or supervisors.
16. When faced with problems, I consider them from different perspectives to gain deeper insights.
17. I actively push for the implementation of new ideas.
18. I am willing to take risks to support new ideas.
19. I find ways to secure the necessary resources to realize new research ideas.
20. I make appropriate plans and strategies to realize new research ideas.
21. Overall, I am a person with a strong spirit of innovation.

#### Research self-efficacy

22. I am confident in my ability to become an outstanding researcher.
23. I possess the motivation and perseverance needed for a researcher.
24. I have a strong interest in engaging in scientific research.
25. My desire to become a researcher is sufficient to help me overcome difficulties in the research process.
26. I am confident that I understand the process of scientific activities very clearly.

### Supervisor support

#### Academic support

27. My supervisor provides advice to help me find the necessary resources.
28. My supervisor guides me in finding relevant literature and research materials.
29. My supervisor teaches me research skills and techniques to assist me in my research.
30. When my research activities require help, my supervisor provides substantial assistance.
31. My supervisor provides timely feedback whenever I submit research results.

#### Emotional support

32. My supervisor is enthusiastic when discussing my research or problems I face.
33. My supervisor is friendly, kind, and approachable.
34. My supervisor appreciates me and makes me feel good about myself and my research work.
35. My supervisor shows respect and values me.
36. My supervisor makes me believe that I can complete my dissertation research.

#### Autonomy support

37. My supervisor encourages me to express my ideas and concerns.
38. My supervisor listens to how I plan to conduct my research.
39. My supervisor encourages me to participate in discussions and respects my ideas.
40. My supervisor gives me some autonomy in my research.
41. My supervisor encourages me to work independently.
